# Disability and severe depression among Peruvian older adults: analysis of the Peru Demographic and Family Health Survey, ENDES 2017

**DOI:** 10.1186/s12888-020-02664-3

**Published:** 2020-05-24

**Authors:** Joshuan J. Barboza, Anderson N. Soriano-Moreno, Anthony Copez-Lonzoy, Josmel Pacheco-Mendoza, Carlos J. Toro-Huamanchumo

**Affiliations:** 1 Universidad San Ignacio de Loyola, Unidad de Revisiones Sistemáticas y Meta-análisis, Lima, Peru; 2 School of Medicine, Universidad Peruana Unión, Lima, Peru; 3 Universidad San Ignacio de Loyola, Unidad de Investigación en Bibliometría, Lima, Peru; 4 Asociación Peruana de Profesionales de las Adicciones – APPADIC, Lima, Peru; 5 Universidad San Ignacio de Loyola, Unidad de Investigación para la Generación y Síntesis de Evidencias en Salud, Av. La Fontana 750, La Molina, Lima, Peru

**Keywords:** Disabled persons, Depression, Mental health, Surveys and questionnaires, Peru

## Abstract

**Background:**

Depression is considered a mental health-related disability that affects approximately 350 million people worldwide. On the other hand, it is estimated that 15% of the world’s population lives with some form of disability, and this scenario is currently riddled with the global burden of mental disorders, non-communicable diseases and other age-related comorbidities.

**Aim:**

To assess the association between disability and depression among Peruvian older adults.

**Methods:**

We used data from the 2017 Peru Demographic and Familiar Health Survey, with a focus on adults aged 50 years and older. Whereas the presence of disability was assessed using different questions of the survey, depression was measured with the Patient Health Questionnaire-9 (PHQ-9). We calculated the adjusted prevalence ratios (aPR) using Poisson regression models with log link function, with their respective 95% confidence intervals (95% CI).

**Results:**

From the study population, 5% had a disability. In addition, 43.3% were screened positive for depression (13.2% for moderately severe/severe). After adjusting for confounding variables, disability was associated with moderate and severe depression (aPR: 1.06; 95% CI: 1.01–1.11, aPR: 1.10; 95% CI: 1.05–1.15).

**Conclusion:**

Disability was positively associated with moderate and severe depression. Public health policies should address the early diagnosis and rehabilitation of patients with any of these problems. Likewise, coping strategies should be promoted among families of persons with disabilities.

## Background

Depression is considered a mental health-related disability that affects approximately 350 million people worldwide [1], causing high healthcare costs due to the recurrence and chronicity of depressive symptoms (DS) [2, 3]. Despite efforts for screening and early interventions, the etiology of depression is still not fully understood [4]. Furthermore, the negative impact of DS on the development and worsening of some non-communicable diseases (NCDs) [5–7] and substance use disorders [8, 9] makes even more difficult the understanding of the already complex pathophysiology.

On the other hand, it is estimated that 15% of the world’s population lives with some form of disability [10], and this scenario is currently riddled with the global burden of mental disorders [11], NCDs [12] and other age-related comorbidities [13]. In addition, people with disabilities usually have to face the loss of role within the family, unemployment, the social and self-stigma of disability and low-quality healthcare [14–18].

Current evidence suggests that there is a close relationship between disability and mental health, which includes an increased risk for depression [1, 19]. However, not only the linked pathophysiology and mechanisms are still not fully understood [4], but there are also several factors (e.g. sociodemographic and clinical) which might act as confounders in this relationship [20, 21].

In Peru, the probability of having a disability and the disability-Adjusted Life Year (DALYs) from depressive disorders increase from the age of 50 years onwards [22, 23]. In addition, whereas a previous study found an association between physical disability and depression in a population aged 60 years and over [24], other population-based studies only focused on mental health and its comorbidities in general [25, 26]. Thus, this study aimed to assess the association between disability and depression among Peruvian adults aged 50 years and older, using the Demographic and Family Health Survey (ENDES 2017).

## Methods

### Source of information

The data used in this research was collected during the 2017 Peru Demographic and Familiar Health Survey (ENDES) [27]. The ENDES is a nationally representative, stratified, cluster sample survey performed annually by the National Institute of Statistics and Informatics (INEI). It is composed of three questionnaires (household, reproductive-age women and health) and is performed with the aim to update knowledge about health indicators of the Peruvian population [28]. For this study, we used the health questionnaire data set, which was applied to one person per household aged 15 or above and selected by the last-birthday method [29]. Databases and additional information about the ENDES methodology are publicly available on the INEI web page.

### Setting and population

During 2017, 36,595 households were surveyed in ENDES and 34,099 individuals participated in answering the health questionnaire. We restricted our analysis to 8298 adults aged 50 years and older [30, 31]. A total of 37 participants (< 0.1%) had missing data in our variables of interest; these were removed from analyses and we worked with a final sub-sample of 8261 individuals.

### Exposure: disability

ENDES assessed disability by using six questions which asked if the responder had permanent limitation to move, talk, heard, see, understand or communicate/interact with other persons. Disability was defined as a positive answer to any of these limitations. Then, it was dichotomized in yes or no.

### Outcome: depression

Depression was assessed using the Patient Health Questionnaire-9 (PHQ-9), which has been previously validated in Peruvian population [32]. The PHQ-9 requires a respondent to report “over the last 2 weeks, how many days have you felt any of the following problems”. Nine items were assessed: (i) “Little interest or pleasure in doing things”; (ii) “Feeling down, depressed, or hopeless”; (iii) “Trouble falling or staying asleep, or sleeping too much”; (iv) “Feeling tired or having little energy”; (v) “Poor appetite or overeating”; (vi) “Trouble concentrating on things”; (vii) “Moving or speaking so slowly that other people could have noticed, or the opposite, being so fidgety or restless that you have been moving around a lot more than usual”; (viii) “Thoughts that you would be better off dead or of hurting yourself in some way”, (ix) “Feeling bad about yourself or that you are a failure or have let yourself or your family down”. The answers of the questions were recoded using a standardized protocol, 0 to 1 day was recoded as ‘0’; 2 to 6 days was recoded as ‘1’; 7 to 11 days was recorded as ‘2’; and 12 to 14 days was recoded as ‘3’.

The total score was divided into the following categories of increasing severity: 1–4 for no significant depression; 5–9 for mild depression, 10–14 for moderate depression; and ≥ 15 for moderately severe/severe depression. These categories were chosen based on the Kroenke et al. research that validated the PHQ-9 for depression severity assessment [33].

### Other variables

Based on previous literature we included as confounder variables: Sociodemographic characteristics (sex, age, education, income level, geographical region), lifestyle habits (daily smoking, harmful alcohol consumption, body mass index) and two comorbidities (diabetes and hypertension).

### Statistical analysis

ENDES data sets were downloaded and imported to R v3.5.2. Using the *survey* package, we specified the complex design using the primary and secondary sample units, weights and strata for each observation. Categorical variables were described using absolute frequencies, and weighted proportions with 95% confidence intervals (95% CI). For assessing the associations between categories of depressive symptom severity (dependent variable) and the presence of disability (explanatory variable), we used a Poisson regression model with log link function and calculated the adjusted prevalence ratios (aPR) with their respective 95% CI. We performed three separate models for each category of depression severity, using the category “no significant depressive symptoms” as reference.

## Results

### General characteristics of the study population

Table 1 shows the characteristics of the sample of 8261 adults aged 50 and older. Nearly 5% of respondents had a disability and 27% were screened positive for depression. Other important variables were hypertension (21.4%), diabetes (8.4%), daily smoking (2.2%), harmful alcohol consumption (0.4%), overweight (41.3%) and obesity (26.8%).

**Table 1:** General characteristics, disability and depression levels of a sub-sample of Peruvian adults, ENDES 2017 (*n* = 8261)

Variables	n	% svy	IC 95%
Sex			
Male	3868	47.8	45.99–49.64
Female	4393	52.2	50.36–54.01
Age group			
50–59	3421	44.4	42.60–46.24
60–69	2476	29.3	27.73–30.91
≥ 70	2364	26.3	24.59–27.94
Education level			
No one	1294	10.4	9.39–11.32
Primary	3654	36.2	34.39–38.07
Secondary	1955	29.1	27.48–30.79
Superior	1358	24.3	22.31–26.23
Geographical Region			
Lima Metropolitan area	797	36.8	33.66–40.03
Rest of the coastline	2290	25.2	22.99–27.32
Highlands	3409	27.1	24.94–29.17
Jungle	1765	10.9	9.76–12.14
Wealth index			
Very poor	3127	20.4	18.97–21.86
Poor	1705	15.9	14.61–17.18
Medium	1323	18.6	17.04–20.11
High	1146	21.6	19.81–23.30
Very high	960	23.6	21.37–25.76
Hypertension			
No	6519	78.6	77.07–80.05
Yes	1742	21.4	19.95–22.93
Diabetes Mellitus			
No	7672	91.6	90.55–92.64
Yes	589	8.4	7.36–9.45
Daily smoking			
No	8078	97.8	97.20–98.34
Yes	183	2.2	1.66–2.80
Harmful alcohol consumption			
No	8232	99.6	99.43–99.84
Yes	29	0.4	0.16–0.57
Body mass index (kg/m2)			
Underweight (< 18.5)	137	1.2	0.88–1.51
Normal weight (18.5–24.9)	3063	30.7	29.05–32.33
Overweight (25–29.9)	3144	41.3	39.50–43.07
Obese (≥30)	1917	26.8	25.09–28.56
Disability			
No	7727	97.8	97.20–98.34
Yes	534	5.0	4.27–5.77
Depressive symptom severity			
No significant depressive symptoms	5609	73.0	71.49–74.56
Mild	1603	16.7	15.41–17.89
Moderate	578	6.1	5.27–6.90
Moderately severe/severe	471	4.2	3.63–4.85

### Depression severity in adults with disabilities

Among people with disabilities, 43.3% had depression (weighted %): 19.4, 10.7 and 13.2% were screened positive for mild, moderate and moderately severe/severe depression, respectively).

### Prevalence of depressive symptom severity by characteristics of the study population

Severe depression was significantly more frequent among women (*p* < 0.001) and respondents from the highlands (*p* < 0.001), considered poor or very poor by their wealth index (*p* < 0.001), with hypertension (*p* < 0.001), with a harmful alcohol consumption (*p* = 0.010), and with a disability (*p* < 0.001). The age group (*p* < 0.001), education level (*p* < 0.001), geographical region (*p* < 0.001) and body mass index (BMI) (*p* < 0.001) were also related to depressive symptom severity (Table 2).

**Table 2:** Depressive symptom severity by characteristics of the study population, ENDES 2017

Variables	Depression levels				***p***
	No significant	Mild	Moderate	Severe^**a**^	
	***n*** = 5609	***n*** = 1603	***n*** = 578	***n*** = 471	
Sex					< 0.001
Male	2979 (77.0%)	575 (14.9%)	170 (4.40%)	144 (3.72%)	
Female	2630 (59.9%)	1028 (23.4%)	408 (9.29%)	327 (7.44%)	
Age group					< 0.001
50–59	2434 (71.1%)	608 (17.8%)	223 (6.52%)	156 (4.56%)	
60–69	1716 (69.3%)	489 (19.7%)	157 (6.34%)	114 (4.60%)	
≥ 70	1459 (61.7%)	506 (21.4%)	198 (8.38%)	201 (8.50%)	
Education level					< 0.001
No one	662 (51.2%)	338 (26.1%)	148 (11.4%)	146 (11.3%)	
Primary	2348 (64.3%)	797 (21.8%)	267 (7.31%)	242 (6.62%)	
Secondary	1464 (74.9%)	320 (16.4%)	111 (5.68%)	60 (3.07%)	
Superior	1135 (83.6%)	148 (10.9%)	52 (3.83%)	23 (1.69%)	
Geographical Region					0.001
Lima Metropolitan area	622 (78.0%)	112 (14.1%)	46 (5.77%)	17 (2.13%)	
Rest of the coastline	1759 (76.8%)	350 (15.3%)	112 (4.89%)	69 (3.01%)	
Highlands	1948 (57.1%)	835 (24.5%)	318 (9.33%)	308 (9.03%)	
Jungle	1280 (72.5%)	306 (17.3%)	102 (5.78%)	77 (4.36%)	
Wealth index					< 0.001
Very poor	1839 (58.8%)	748 (23.9%)	284 (9.08%)	256 (8.19%)	
Poor	1123 (65.9%)	338 (19.8%)	131 (7.68%)	113 (6.63%)	
Medium	956 (72.3%)	243 (18.4%)	66 (4.99%)	58 (4.38%)	
High	875 (76.4%)	174 (15.2%)	65 (5.67%)	32 (2.79%)	
Very high	816 (85.0%)	100 (10.4%)	32 (3.33%)	12 (1.25%)	
Hypertension					< 0.001
No	4578 (70.2%)	1203 (18.5%)	421 (6.46%)	317 (4.86%)	
Yes	1031 (59.2%)	400 (23.0%)	157 (9.01%)	154 (8.84%)	
Diabetes Mellitus					0.136
No	5233 (68.2%)	1476 (19.2%)	534 (6.96%)	429 (5.59%)	
Yes	376 (63.8%)	127 (21.6%)	44 (7.47%)	42 (7.13%)	
Daily smoking					0.002
No	5463 (67.6%)	1577 (19.5%)	569 (7.04%)	469 (5.81%)	
Yes	146 (79.8%)	26 (14.2%)	9 (4.92%)	2 (1.09%)	
Harmful alcohol consumption					0.008
No	5597 (68.0%)	1590 (19.3%)	576 (7.00%)	469 (5.70%)	
Yes	12 (41.4%)	13 (44.8%)	2 (6.90%)	2 (6.90%)	
Body mass index (kg/m2)					< 0.001
Underweight (< 18.5)	84 (61.3%)	21 (15.3%)	10 (7.30%)	22 (16.1%)	
Normal weight (18.5–24.9)	1982 (64.7%)	642 (21.0%)	215 (7.02%)	224 (7.31%)	
Overweight (25–29.9)	2205 (70.1%)	582 (18.5%)	218 (6.93%)	139 (4.42%)	
Obese (≥30)	1338 (69.8%)	358 (18.7%)	135 (7.04%)	86 (4.49%)	
Disability					< 0.001
No	5343 (69.1%)	1483 (19.2%)	517 (6.69%)	384 (4.97%)	
Yes	266 (49.8%)	120 (22.5%)	61 (11.4%)	87 (16.3%)	

^**a**^Included both moderately severe and severe depression

### Association between disability and depressive symptom severity

Table 3 shows the prevalence ratios after adjusting for sex, age, education, income, BMI, smoking, harmful alcohol consumption, diabetes and hypertension. We found that disability was positively associated with moderate and severe depression (aPR: 1.06; 95% CI: 1.01–1.11 and aPR: 1.10; 95% CI: 1.05–1.15, respectively).

**Table 3:** Adjusted prevalence ratios (aPR) for depressive symptom severity, according to disability, ENDES 2017

Depressive symptom severity	Disability	aPR^**a,b**^	95% CI	***p***
Mild^c^	No	Ref		
	Yes	1.03	0.97–1.08	0.376
Moderate^c^	No	Ref		
	Yes	1.06	1.01–1.11	**0.025**
Moderately severe/severe^c^	No	Ref		
	Yes	1.10	1.05–1.15	**< 0.001**

^a^ Prevalence ratio was adjusted for sex, age, education, income, BMI, smoking and harmful alcohol consumption, diabetes and hypertension

^b^ PR calculated using Poisson regression with a robust error variance

^c^ Reference category = no significant depressive symptoms

## Discussion

### Main findings

We conducted a secondary analysis using data from the 2017 Peru Demographic and Family Health Survey. The overall prevalence of severe depression in the population was 4.2% and among people with disabilities was 13.2%.

### Depression and disability

Some mechanisms could explain the reciprocal relationship between disability and depression. First, depression itself is a disabling illness [34]. Second, disability is an independent determinant of the severity of depressive symptoms in different health conditions [34, 35]. Third, depression is usually associated with other important comorbidities such as hypertension and diabetes [36], which could result in a complex disabling condition. Fourth, disability could play the role of chronic stressful condition which increases the risk of developing depression [19]. Fifth, disability and depression might share hormonal and metabolic pathways: Depression has been linked with high levels of cortisol [37] but it has been hypothesized that physical exercise could modulate these levels possibly due to an upregulation of the glucocorticoid receptor [38]. Since physical inactivity is particularly prevalent among adults with disabilities [39], depression could be a reflection and a consequence of this scenario. Sixth, the complex social and family context of disability can increase the severity of depression [40–42].

It was estimated that in 2015 the proportion of the world’s population with depression was 4.4%. Prevalence rates vary by age and peak in most adulthood (above 7.5% in women aged 55–74 and above 5.5% in men) [1]. Depression contributes to functional disability in patients with chronic medical conditions and leads to impairment in self-maintenance and instrumental activities of daily living [43]. Unfortunately, the diagnosis can be particularly tricky because of the clinical overlap of several symptoms that may confound the complex clinical picture of this disorder [44], especially among people with disabilities [45, 46].

In Peru, one study that assessed the relationship between depression and disability was conducted by Martina M et al. [24]. The research team described that people with disabilities and some level of depression represented 12.7% of the surveyed population. However, several confounding variables were not assessed, and the study population was restricted to people ≥60 years, which limited the extrapolation of the results. A study conducted in Italy by Solaro C et al. (2016) [47] reported that 19% of the adult population with multiple sclerosis (considered in this context as a disability) had severe depression. The authors attributed this finding to the correlation between brain damage and the frequency of depressive symptoms, more critical than disability per se. Hughes R et al. (2007) found that 75.4% of US rural women with a physical disability had severe depression [48], a much higher proportion than in our study. However, both of them used the Beck Depression Inventory-II instead of the PHQ-9. Although in our study mild depression was the most prevalent, it is necessary to consider that disability (primarily physical) in adults is often long and permanent. Thus, depression might reach higher levels if psychological and psychosocial interventions are not taken [1].

### Public health relevance

Depression is considered the leading cause of disability worldwide [49]. In Peru, the implementation of mental health care is still an unmet challenge. It is not available in several regions, and private health insurance is not required by law to cover such care [50]. In addition, the absence of a community-based care and rehabilitation system forces patients with disability and depression to live and stay all day at their homes. This often results in family issues, attrition of the primary caregiver, and social discrimination for both the individuals and their families [40–42, 50, 51].

In Peru, the Mental Health Law (N° 30,947) was recently promulgated on April 2019, establishing the legal framework to guarantee access to services, promotion, prevention, treatment and rehabilitation in mental health, as conditions for the full exercise of the right to health and well-being of the individual, the family and the community [52]. This is certainly an important beginning in the process of addressing the burden of mental illness in the country, especially because it includes the adoption of measures to eliminate barriers to access to mental health care in people with disabilities. However, it is necessary to assess the impact and usefulness of this law, and our results could serve as a baseline for future studies.

### Strengths and limitations

This was a population-based study with a representative and multi-stage sampling. In addition, since ENDES is based on DHS methodology, our results could be compared with other surveys either in Peru or other countries. However, some limitations should be highlighted. First, we cannot infer causality in the interpretation of results due to the cross-sectional design of the study. Moreover, in the interpretation of the results reverse causality can not be ruled out. Second, the World Health Organization defines disability as a continuous phenomenon; however, this variable is dichotomized in the ENDES 2017 (yes/no). Third, since this was a secondary analysis we could not include some potential confounding variables such as the family history of depression or other comorbidities different from hypertension and diabetes. Fourth, PHQ-9 is a screening and not a diagnostic tool for depression, which could produce false-positive results; however, we used validated cut-off points for depressive symptoms severity, which should reduce the risk of misclassification.

## Conclusion

This study found that disability was associated with moderate and severe depression. It is essential to prioritize public health policies that address the early diagnosis and rehabilitation of patients with any of these problems. Likewise, coping strategies should be promoted among families of persons with disabilities.

## Data Availability

The datasets generated and/or analyzed during the current study are available in the Peruvian National Institute of Statistics and Informatics repository: http://iinei.inei.gob.pe/microdatos/

## Abbreviations

ENDES: Encuesta Demográfica y de Salud Familiar (Demographic and Family Health Survey)
INEI: Instituto Nacional de Estadística e Informática (National Institute of Statistics and Informatics)
PHQ-9: Patient Health Questionnaire-9
BMI: Body mass index
PR: Prevalence ratio
95% CI: 95% confidence intervals
NCDs: Non-communicable diseases
DS: Depressive symptoms
DALYs: Disability-Adjusted Life Year
DHS: Demographic and Health Survey
